# Collaborative coding in inductive content analysis: Why, when, and how to do it

**DOI:** 10.1002/jgc4.70030

**Published:** 2025-04-30

**Authors:** Free Coulston, Fiona Lynch, Danya F. Vears

**Affiliations:** ^1^ The University of Melbourne Parkville Victoria Australia; ^2^ Murdoch Children's Research Institute Parkville Victoria Australia; ^3^ School of Medicine Deakin University Waurn Ponds Victoria Australia; ^4^ Centre for Biomedical Ethics and Law KU Leuven Leuven Belgium

**Keywords:** co‐coding, collaborative coding, content analysis, inductive analysis, methods, qualitative research

## Abstract

Inductive content analysis (ICA) is a useful method for analyzing qualitative data in genetic counseling research. It is particularly relevant when the goal is to examine and improve practices or develop recommendations. Although ICA can be undertaken by a single analyst, ideally there is involvement of multiple analysts (or co‐coders). Co‐coding can bring many benefits to qualitative analysis that sits within a constructivist paradigm, including developing a representation of the data that is not only understandable to more than one individual but also richer and more nuanced. It also provides an opportunity for mentoring more junior researchers and can be an efficient way to analyze large datasets. However, co‐coding requires important planning and consideration, and there is currently a paucity of clear guidance. In this paper, we provide an outline of the small body of existing literature on this topic and propose six flexible step‐by‐step components of our approach to co‐coding in ICA, based on our own work. We have utilized it to analyze reporting practices and perspectives for diagnostic genomic sequencing, informed consent for genetic testing, data sharing and storage, and genomic newborn screening, among other topics. To illustrate these components, we present some example vignettes to show how these procedures can be applied in different scenarios and with different analysts.

**What is known about this topic:**

Inductive content analysis is a useful method for analyzing qualitative data in genetic counseling research. Guidance on involving multiple analysts in inductive content analysis is lacking.

**What this paper adds to the topic:**

This paper proposes six flexible step‐by‐step components for co‐coding in ICA. Example vignettes are provided to illustrate how these components can be applied in different scenarios and with different analysts.

## 1 INTRODUCTION

The field of qualitative research is becoming more established, and qualitative research methods are increasingly being recognized as an important approach to exploring a range of questions across health‐related areas (Renjith et al., 2021). We (the authors) have previously proposed that inductive content analysis (ICA) is very well suited to answer many research questions in health‐related qualitative research (Vears & Gillam, 2022). This is because ICA is an exploratory data analysis approach, making it appropriate when little is known about the topic under investigation. Additionally, the results of ICA remain close to the data (rather than abstract concepts derived from using theory) making it useful for the development of practical outcomes or recommendations.

For these reasons, ICA can also be a valuable analytical method in the field of genetic counseling (Lynch, Gillam, et al., 2023); a considerable amount of genetic counseling research is aimed at assessing and improving practices and services. For example, we have (collectively) used ICA to analyze reporting practices and perspectives for diagnostic genomic sequencing (Vears et al., 2019; Vears, Borry, et al., 2020); informed consent for genetic testing (Phillips et al., 2020; Vears et al., 2017a, 2017b; Vears et al., 2020b); data sharing and storage (Lynch et al., 2024a, 2024b); and genomic newborn screening (Cao et al., 2022; Lynch et al., 2023a, 2023b).

Our previous publication described in detail when it might be appropriate to use ICA and outlined the steps involved (Vears & Gillam, 2022). This paper has been highly cited across a range of different areas and parts of the world (Ahmad‐Kamil et al., 2024; Allen et al., 2024; Appiah et al., 2023), suggesting it has met a need for a clear description of how to undertake this approach. We have since received requests for further information about when and how to involve multiple researchers in the coding process; we refer to this as “co‐coding.” As we will demonstrate, very few publications have clearly described the rationale and process of involving multiple coders in ICA. To address this gap in the literature, this article aims to outline and describe different approaches to co‐coding that can be used in ICA based on existing literature and our own experience.

### 1.1 Brief overview of ICA

As the name suggests, ICA is an inductive approach to analyzing qualitative data. By inductive, we mean that the codes are developed during the coding process from the data itself, rather than having a predetermined coding schema (which is known as a deductive approach). Coding takes place in rounds; first, labels known as categories or big picture meaning units are applied to larger segments of the text, and then second, the segments of text within these categories are coded in finer detail into subcategories. Unlike thematic analysis, interpretation generally remains quite close to the data, rather than applying theoretical frameworks with which to develop a deeper understanding of the phenomenon under investigation. For a detailed description of the process of using ICA, please see Vears and Gillam (2022).

#### 1.1.1 Terminology

Although ICA can be undertaken by a single analyst, ideally there should be some element of co‐coding. This is because ICA is an inductive approach and some degree of interpretation is involved. Co‐coding can have several benefits, including obtaining multiple perspectives of data interpretation to enrich the results and enhance rigor. Our reading suggests that the terms “consensus coding,” “co‐coding,” and “collaborative coding” are all used to describe the involvement of multiple analysts in qualitative research. Here, we outline this different terminology, while recognizing that these terms are sometimes used interchangeably.

The term “consensus coding” is often used to describe coding reliability approaches. These approaches sit within a positivist paradigm and are focused on multiple analysts reaching high interrater (intercoder) reliability in their coding (Braun & Clarke, 2021a, 2021b). However, ICA (as we conceptualize it) sits within a constructivist paradigm (the belief that we mentally construct the world around us (Lincoln & Guba, 2013)). Constructivism focuses on the interpretation of data, rather than passive absorption of information. It aims to derive meaning rather than seek an absolute truth. It is for this reason that the goal of co‐coding in constructivism is *not* to establish an objective truth about the phenomenon being studied, but to construct a broader and deeper understanding by drawing on more than one individual interpretation.

ICA acknowledges that we each view the world through our own lens, which is informed by our past experiences and knowledge. This means that, on first pass, our analysis is quite likely to be different from that of another researcher. Therefore, we propose that traditional coding reliability methods are not appropriate to use with ICA because we recognize that each of our ways of analyzing qualitative data might be “correct.” For this reason, this paper will focus on exploring other ways to involve multiple analysts. More information about consensus coding within a positivist paradigm can be found in the Ahuvia (2001) and Downe‐Wamboldt (1992) texts.

The terms “co‐coding” and “collaborative coding” are associated with a range of different qualitative analytical approaches, including grounded theory (Pieters & Dornig, 2013), thematic analysis (Richards & Hemphill, 2018), and inductive content analysis (Dehmoobad Sharifabadi et al., 2019). These terms generally relate to the process whereby multiple analysts code the same dataset to derive a richer understanding of the meaning of the data, rather than to achieve high intercoder reliability (Byrne, 2022). To make this distinction clear, we will adopt the term used by Flicker and Nixon (2015) of developing a “shared understanding” of the dataset (p. 620) to describe the bringing together of multiple perspectives during co‐coding in ICA.

It should be noted from the outset that co‐coding in ICA is more than a “sense‐check” by a critical peer who is external to the project; we will explain why this distinction is important as we delve deeper into the discussion (Byrne, 2022). Henceforth, we will use the term “co‐coder” to describe a researcher who is working within a team of two or more people to analyze a data set, differentiating this from researchers acting as “critical peers,” “sense checkers,” or “auditors” where they are reviewing and providing feedback on the findings of the analysis but not actually performing any coding themselves.

#### 1.1.2 Why co‐code in qualitative research?

As we see it, there are three main ways in which co‐coding is beneficial in ICA. First, by asking multiple researchers to code (at least some of) the data within a project, we are aiming to develop a representation of the data that is not only understandable to more than one individual but also richer and more nuanced because it includes the interpretation looked at through multiple lenses (Flicker & Nixon, 2015; Gregor et al., 2020; Richards & Hemphill, 2018). This aligns with the work by Lincoln and Guba that discusses researcher triangulation as one of the criteria for trustworthiness in qualitative research (Lincoln & Guba, 1986). Second, co‐coding in ICA is practical in nature; it can assist researchers who have large datasets in managing the data more efficiently (Flicker & Nixon, 2015; Richards & Hemphill, 2018). It can also empower researchers who are part of larger teams to feel a greater sense of responsibility or ownership of the project when they are entrusted with analyzing a subset of the data (Flicker & Nixon, 2015). Third, as we discuss in Vignettes 1 and 2 below, co‐coding can be an effective way of training beginning researchers to use ICA (Richards & Hemphill, 2018).

#### 1.1.3 Previous descriptions of co‐coding in inductive content analysis

Published literature relating to content analysis demonstrates that there is some variation in what authors consider to be the exact purpose of co‐coding. Polit and Beck (2004) suggest that co‐coding of at least some of the transcripts in a dataset is necessary to ensure intercoder reliability, which Ahuvia (2001) and Graneheim and Lundman (2004) suggest is helpful for assessing the coding quality and is a method for assessing credibility, respectively.

Yet, authors have questioned the appropriateness of researchers assessing agreement as a form of validation, such as Sandelowski who cites the subjectivity in interpretations leading to multiple versions of reality (1993, 1998). Schamber (2000) too highlights the dependence of the coding achieving reliable results on the training and experience of the researchers. Downe‐Wamboldt (1992) also flag “fatigue, personal bias and perception” as issues in coding, suggesting that these lead to “human error” (p. 319).

More fittingly, Ahuvia (2001) describes two broad types of content analysis and suggests that different degrees of co‐coding are appropriate for each type. First, “traditional” content analysis (which they associate with counting) is not compatible with co‐coding because it requires calculations of intercoder reliability. Second, instead of intercoder reliability, interpretive content analysis uses public justifiability (which means submitting the texts being analyzed, the codes, and potentially a justification of the codes, along with the manuscript to the journal), and therefore multiple coders are recommended; Ahuvia suggests this should be done collaboratively rather than independently. This second approach appears to align most closely with our definition of ICA.

#### 1.1.4 Previous descriptions of co‐coding in inductive thematic analysis

Due to the limited literature describing co‐coding in ICA, we will also discuss methods used in inductive thematic analysis. As Braun and Clarke (2021a) note, inductive content analysis often sits as a “close cousin” (p. 203) of thematic analysis approaches, making it an appropriate body of literature from which to draw concepts that are relevant and applicable to ICA.

Two potentially relevant descriptions of co‐coding methods from within the inductive thematic analysis literature are “Collaborative Qualitative Analysis” as described by Richards and Hemphill (2018), and Flicker and Nixon's (2015) “DEPICT model” of collaborative qualitative data analysis. The “Collaborative Qualitative Analysis” method was developed to provide a structured approach to thematically analyzing qualitative data in teams. The “DEPICT model” was developed to guide analysts working in Community‐Based Participatory Research on how to collaboratively code with a wide variety of interested parties (including community members). Many other publications (e.g., Morse et al., 2023; Ohta et al., 2024; Surratt et al., 2023), although not citing specific methodological frameworks, describe co‐coding methods that align very closely to those outlined above. The steps of these methods, alongside our proposed approach for co‐coding in ICA, are outlined in Table 1.

**TABLE 1:** Frameworks describing collaborative coding in inductive thematic analysis, and their alignment with co‐coding in ICA.

Approaches described in inductive thematic analysis		Proposed approach for co‐coding in ICA
“Collaborative qualitative analysis” (Richards & Hemphill, 2018)	“The DEPICT model” of collaborative qualitative data analysis (Flicker & Nixon, 2015)	
Step 1 (*preliminary organization and planning*): Research team meets to plan analysis	N/A	a) Planning for collaborative analysis
Step 2 (*open and axial coding*): Each analyst reads 2–3 transcripts Analysts are encouraged to make memos as they read	D) (*dynamic reading*): Each analyst reads a subset of the transcripts (transcripts allocated by someone familiar with whole dataset)	b) Familiarization with project aims, relevant literature, and dataset
Step 2 (*open and axial coding*): Independent open‐coding of the allocated transcripts using analysts' preferred method	D) (*dynamic reading*): *Research questions are kept close at hand, and analysts are encouraged to make memos as they read*	c) Initial coding
Step 2 (*open and axial coding*): Team discussion of codes and memos. Step 2 repeats until team “when all coders feel comfortable with advancing to the development of a codebook”—after approximately 30% of transcripts have been coded Step 3 (*development of a preliminary codebook*): One analyst develops a draft codebook based on the results of Step 2. The codebook is reviewed by all analysts and refined	E) (*engaged codebook development*): as a group, analysts c*reate preliminary codebook from clustering memos, and defining subsequent categories*	d) Coming to “shared understanding” of codes/ categories
Step 4 (*pilot testing the codebook*): All analysts proceed to code an additional 2–3 transcripts using the codebook. The codebook is further refined through team discussions Step 5 (*the final coding process*): The entire dataset (including previously coded transcripts) is coding using the final codebook	E) (*engaged codebook development*): Codebook is piloted and refined by *team members code several transcripts and then discuss how well the framework was able to capture the range of perspectives within the data set* P) (*participatory coding*): The entire dataset (including previously coded transcripts) is coding using the final codebook. The dataset is split between analysts, with each transcript coded by a minimum of two analysts	e) Subsequent coding
Step 6 (*reviewing the codebook and finalizing themes*): The analysts meet and use the codebook to inform development of *a thematic structure comprised of themes and associated subthemes that forms the basis for the results in the manuscript*	I) (*inclusive reviewing and summarizing of categories*): Analysts are paired together and develop a summary of each category C) (*collaborative analysis*): The summary pages are integrated to form draft results. The wider research team meets to contextualize the draft results within the key elements of the project	f) Development of final categories (results)
	T) (*translating*): Creation of a dissemination plan	

## 2 A PROPOSED APPROACH FOR CO‐CODING IN ICA

**FIGURE 1:**
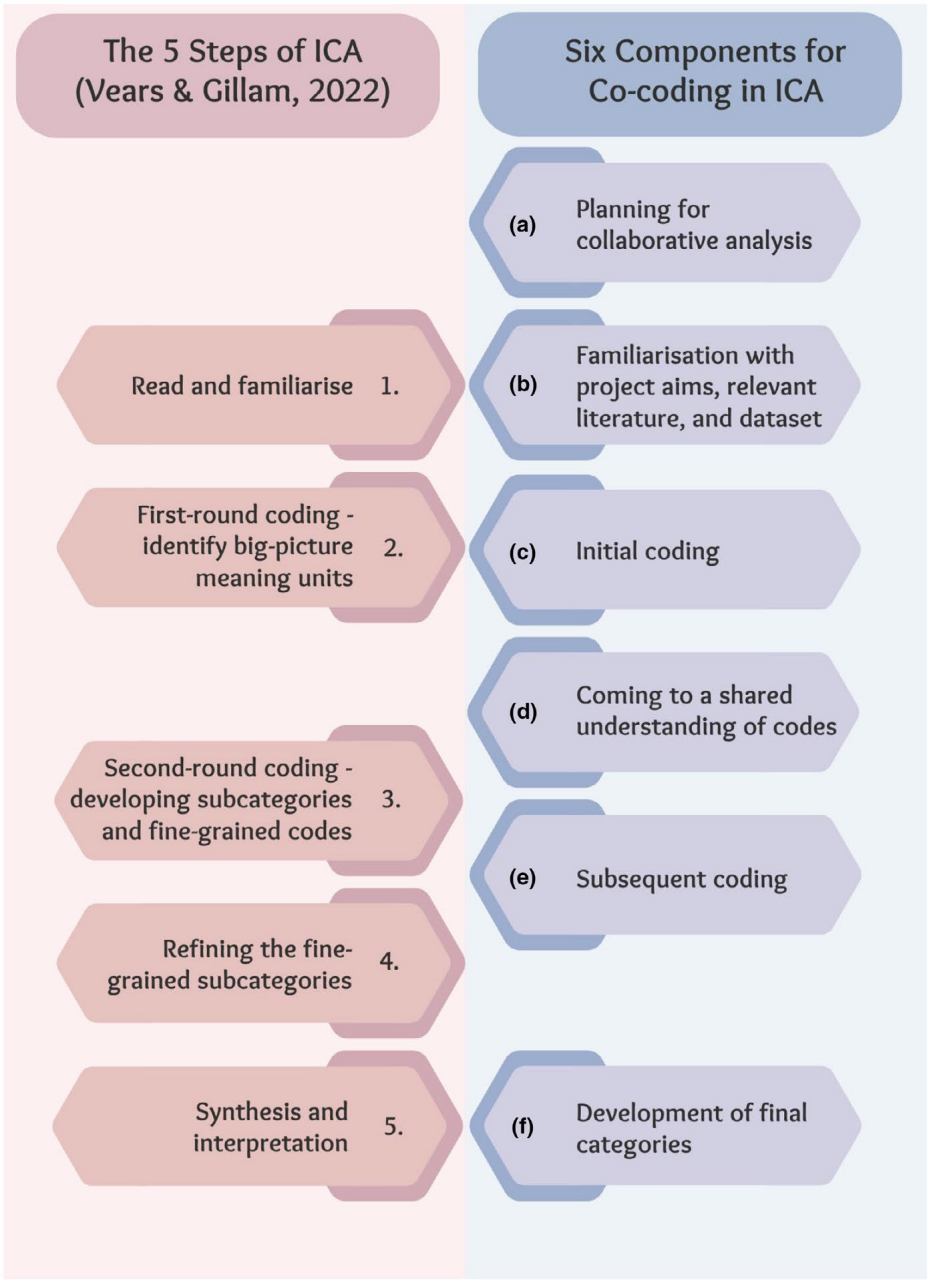
The alignment between the Steps of ICA (Vears & Gillam, 2022) and the co‐coding in the ICA approach.

This section aims to provide guidance and suggestions for undertaking co‐coding in ICA, acknowledging that each project will need to tailor the approach to suit its research question, data, and group of analysts, and this should be clearly described in any presentation or publication of the research. Although our approach outlines six distinct components, like all qualitative analysis processes, there will be iterative movement across and between components. Figure 1 illustrates how this proposed co‐coding approach aligns with Vears and Gillam's (2022) original description of ICA.

### 2.1 Component (a): Planning for collaborative analysis

Planning for the co‐coding process in ICA involves considering how many co‐coders to include, who they will be, and how you will approach co‐coding. It is also important to consider and make explicit your dissemination plan (including publication and other knowledge translation strategies), including how the co‐coders will be represented.

#### 2.1.1 How many co‐coders?

When considering the number of analysts involved, it is important to be aware of some of the potential challenges. The bringing together (physically or virtually) of multiple analysts, keeping timelines on track, and the complexity of integrating multiple perspectives respectfully and collaboratively are challenges described in the literature and echoed in our own experiences (Richards & Hemphill, 2018).

In our own work, we typically have two or three researchers co‐coding. Two to four co‐coders can strike the balance between diverse perspectives bringing richness to the analysis, while reducing the burden of coordinating a large team and the time needed to integrate and synthesize the various interpretations of the data (Richards & Hemphill, 2018). This is not to say that having more than four coders is not possible or desirable, particularly if it is a large‐scale project: Just that this does increase the complexity.

#### 2.1.2 Who are the co‐coders?

When considering who will make up the analysis team, co‐coders should be selected carefully while considering what the goal is that you are trying to achieve. Is the goal:

- To educate a more junior researcher in how to undertake ICA? If so, the co‐coding process will be a mentoring exercise. Although the analysis will benefit from the junior researcher's perspective, a senior researcher will need to be prepared to take a supervisory role. You should factor in additional time for the analysis to allow for the training component.
- To bring multiple perspectives to the interpretation? If so, you will need to consider the different resources required to co‐code with established qualitative researchers versus those without qualitative research experience. You also need to consider the experiences and backgrounds of those you are enlisting to co‐code. For example, including the perspectives of other knowledge holders (such as people with clinical, professional, or lived experiences) can enrich your analysis and interpretation through drawing on diverse social identities and their intersections (positionality) in the construction of knowledge.
- To use a collaborative approach to reduce the burden on the individual researcher? In this case, you may decide to choose a peer with considerable experience in the ICA method to enhance efficiency.

Some people may be better suited to roles other than co‐coder within the analysis team, such as “critical peer” or “sense‐checking” roles to enhance credibility, or in the final stages of creating a framework or model from the resulting categories. Whoever makes up your analysis team, it is key that all co‐coders are aligned on:

- the research question(s) or aim(s);
- the underlying analysis method (e.g., ICA);
- the reason behind using a co‐coding approach;
- timelines and meetings, for example, the amount of time to be spent on each component of co‐coding, whether team meetings will be held synchronously or asynchronously, and ensuring that these are scheduled well in advance;
- how the multiple perspectives will be brought together into a shared understanding of the analysis (this will differ depending on whether you are coding in teams of peers, vs an experienced analyst mentoring a newer researcher); and
- the expectations around remuneration, acknowledgement, and/or coauthorship.

#### 2.1.3 How will you co‐code?

Before coding begins, the units within the dataset (e.g., interview transcripts) will need to be distributed to the co‐coders, ideally by someone familiar with the entire dataset (e.g., the interviewer) so that each coder receives a variety of participant perspectives. Allocations may be distributed either exclusively to one co‐coder or overlapped so that each unit of data is coded by two (or more) different analysts.

An important consideration is whether the team will use data management software (such as NVivo), word processing software (such as Microsoft Word), or hard copies of the data for analysis. We have found virtual data management methods (e.g., either NVivo or a cloud‐based document) are a key aspect of easily sharing elements of the analysis with co‐coders. However, everyone needs to have access to the same program, and the results of coding should be shareable. Investigate co‐coders' access rights from the outset.

### 2.2 Component (b): Familiarization with project aims, relevant literature, and dataset

As with all ICA, time must be allocated for analysts to become deeply familiar with the research question/s and the key aims of the project to have a clear idea of the need or gap that the research is addressing. Depending on the project and coders' experience, it may also be important for them to be familiar with the key literature to provide context for code development. Some suggestions for achieving this are asking analysts to read the study protocol or providing a forum (either virtually or in‐person) for presenting and discussing these key elements.

Familiarization with the dataset itself is also important. As described in Step 1 of the ICA method (Vears & Gillam, 2022), this is usually achieved by repeated listening to recordings and/or reading of transcripts while making notes or memos of initial impressions of the data. In the co‐coding approach, exactly how this occurs depends on how your team answers the following questions:

- How much of the data should the analysts be familiar with (e.g., just the transcripts they are coding or the whole dataset)? Co‐coders involved in the interpretation step of the analysis will likely benefit from greater familiarity with a larger portion or entirety of the dataset.
- Should you be together (virtually or in‐person) when familiarizing yourself with the data to encourage reflections, or is it more beneficial to familiarize and reflect individually? This comes down to personal preference.

### 2.3 Component (c): Initial coding

Here, the analysts will complete Step 2 of ICA: first‐round coding (Vears & Gillam, 2022). This involves labeling sections of the text that relate to concepts relevant to the research question, also known as “big picture” meaning units. This is different from line‐by‐line coding, which is much more detailed and happens in the second‐round coding step. Big picture codes are similar to themes but differ in that they are not developed with the intention of being extrapolated further using theoretical frameworks. With regard to co‐coding, this initial coding step can occur in multiple ways. Analysts may decide to code the data allocated to them individually and then come together to discuss their codes to develop a “shared understanding” (Component (d). This may be more appropriate if there is no overlap between transcript allocations. Alternatively, the co‐coders may want to co‐code together, although the need to schedule co‐coding sessions can take time. These decisions will depend on considerations such as analyst experience, data complexity, and financial resourcing for the analyst's time, which should be discussed in Component (a).

### 2.4 Component (d): Coming to a shared understanding of codes

In this component, the analysis team aims to develop a refined list of codes (“coding schema”) to be used by all co‐coders in Component (e). The “coding schema” is a high‐level, broad framework that describes the overall structure and organization of codes within the big‐picture categories. Other terms such as “coding tree” (Vears & Gillam, 2022) “coding frame,” or “codebook” (Braun & Clarke, 2021b) are also used in the literature to describe similar concepts but tend to provide more granular‐level detail of each code, including a formalized definition, the codes inclusions and exclusions (Turner‐Essel & Ryan, 2023), and may be set a priori rather than developed inductively, as is integral to ICA. Building and documenting a shared understanding of the big‐picture categories is important to bring together the co‐coders' interpretations. We are not aiming for “agreement” on a final single “truth” (as would be the goal of a positivist approach) but rather engaging in discussion (often iteratively) to provide richer and more complex, and nuanced meaning across the preliminary analysis (Byrne, 2022; Flicker & Nixon, 2015). Others have described how this process plays out in practice, most commonly through discussions with another researcher (Morse et al., 2023) or the whole analysis team (Shea et al., 2008). Bringing the whole co‐coding team together for two or three meetings is a good way to achieve the goals of this stage. In some projects with more experienced and “in‐synch” analysis teams who have worked together before and know that their coding is generally well aligned, both first‐ and second‐round coding may have been completed prior to meeting.

### 2.5 Component (e): Subsequent coding

Following the development of a shared understanding of the coding schema, co‐coders will use the “big‐picture” coding schema developed in Component (d) to recode the data. This component is an additional activity between ICA Steps 2 and 3. After this recoding takes place, co‐coders can proceed to the second‐round coding and then refine the fine‐grained subcategories (i.e., ICA Steps 3 and 4 (Vears & Gillam, 2022)). Again, this can be done in multiple ways, but doing these two steps independently, before bringing the team together for one or two meetings to finalize this component, often works best.

### 2.6 Component (f): Development of final categories

This involves the synthesis and interpretation of the data, which aligns with ICA Step 5 (Vears & Gillam, 2022). We have typically brought together the whole team for two or three meetings, and the richness that each analyst brings to the interpretation is often wonderfully apparent in this stage. We have seen strong leadership and facilitation of this stage by the project lead as they often have that “satellite view” of the project as a whole. Practical considerations (such as journal publication requirements) may also shape this step.

## 3 EXAMPLES OF CO‐CODING IN ICA IN PRACTICE

In this section, we present three vignettes that describe different ways that co‐coding in ICA could be approached based on our various experiences and the considerations above.

### 3.1 Vignette A: Co‐coding with an experienced qualitative researcher and a graduate research student

DV often supervises students across Honors, Masters, and PhD levels, as well as supervising interns for work placements. One project involved an Honors student who had completed interviews with 12 members of the Vietnamese community in Australia about their views on participating in genomics research.

#### 3.1.1 Component (a): Planning for collaborative analysis

For this project, DV was the student's primary supervisor and took the lead with assisting the student in their analysis. As this was not a very large study, we felt having two co‐coders was sufficient. The student's other two supervisors acted as “sense checkers,” providing content expertise but not participating in the analysis. The study team also consisted of a community coresearcher who was not involved in the analysis but to whom the results were presented prior to publication to assess how aligned the findings were with their experiences as part of the Vietnamese community.

The team identified DV as the most appropriate co‐coder for the student because of her leadership role on the wider project, her content knowledge, and her methodological experience. DV and the student decided to use NVivo to manage the co‐coding due to familiarity and organizational access. DV allocated plenty of time to the project knowing that the student was new to qualitative research methods and would need support during their training.

#### 3.1.2 Component (b): Familiarization with project aims, relevant literature, and dataset

Having completed a literature review prior to starting data collection, the student was very familiar with the topic. As the student conducted the interviews, DV was less familiar with the data at the outset of analysis. However, DV was heavily involved in the design of the interview guide, so she was aware of what was being covered in the interviews. She was also familiar with the literature in the field. As such, it was decided that DV would only familiarize herself with the interviews that she was going to analyze, rather than the entire dataset.

#### 3.1.3 Component (c): Initial coding

The student completed the initial round of coding. However, after the student had coded half of an interview transcript, DV independently (i.e., without looking at the student's codes) co‐coded the same half of the transcript. The student then compared their big picture codes with those of DV and any discrepancies were discussed between the two co‐coders. This involved each researcher explaining their rationale for the particular way they had coded the text. The aim was to reach a shared understanding of what was being expressed in the data and to find a label for it that both researchers believed represented this concept. The outcome of this discussion was a slightly revised preliminary coding schema which the student then used to code the remainder of the transcripts. DV independently co‐coded another transcript, as well as checking the coding of one other transcript coded by the student, which involved comparing the student's codes with the transcript and making sure that DV understood the rationale behind the coding and believed them to be representative of the transcript. As the coding completed by both the student and DV was very similar, no other transcripts were co‐coded at this stage. However, if there had been any further divergence, DV would have co‐coded more of the dataset.

#### 3.1.4 Component (d): Coming to a “shared understanding” of codes

While this student was analyzing their data, another Honors student was analyzing the data from a parallel set of interviews they had conducted with members of the Filipino community. As the questions across the two sets of interviews had been virtually the same, and as the supervisory team was the same across the two projects, the team felt that this phase of developing a shared understanding of the codes would be best done as a team with people from both projects. In this meeting, the students presented their coding schema (i.e., the list of big picture content categories) to the research team, who provided feedback based on their expertise. Each student did this by talking the team through an entire transcript (and its associated higher order codes), so that everyone could discuss how well the codes represented the data that was being coded. There was not one “right” answer; the intention was to enrich the overall understanding of the transcript.

#### 3.1.5 Component (e): Subsequent coding

After a shared understanding was reached, the student conducted second‐round coding on all of the transcripts. As in the first round of coding, DV checked the second‐round coding of the first half of a transcript to make sure the student was on the right track before they coded the entire dataset. DV also conducted independent second‐round coding on a different transcript than that which had been analyzed in the first round of coding. Again, the student compared their coding with that of DV, and any discrepancies were discussed. At the end of this process, the student presented the overall coding schema to DV. Discussion of the coding schema made them realize that a further round of coding was required on one of the big picture categories, which the student did subsequently. The student presented the entire coding schema to the research team again for feedback.

#### 3.1.6 Component (f): Development of final categories

In the interpretation phase, the student worked both independently and also with the other student to develop an interpretation of the results. As part of the interpretation phase, the student also presented the overall findings to the community coresearcher to gain their insights into how well the findings represented their understanding of their community and to try to explain some of the findings that appeared to be very culturally specific. DV and the other supervisors reviewed the student's thesis draft prior to submission.

### 3.2 Vignette B: Co‐coding with an experienced qualitative researcher and multiple professional entry‐to‐practice students

FC led a project that utilized ICA to analyze physiotherapy students' and supervisors' experiences of a novel experiential intervention prior to clinical placement. The data consisted of transcripts of four focus groups (two with groups of physiotherapy students and two with supervisors), all of which FC had facilitated.

#### 3.2.1 Component (a): Planning for collaborative analysis

In this scenario, many of the considerations for this component were nonnegotiable (such as “how many” co‐coders), as this project was part of a compulsory research subject in the final year of an entry‐to‐practice physiotherapy course. The students were undertaking ICA for the first time, and the co‐coding process was therefore education‐focused. As such, FC had allowed for additional time to guide and mentor the students through the analysis. FC took the role of analysis lead, providing the students with a clear step‐by‐step guide of how they would utilize the co‐coding process d (as described below), including timelines. She also provided resources on the underlying analysis method (in this case, ICA as described by Vears & Gillam, 2022), as well as the benefits and challenges of involving multiple analysts. She was clear with the students upfront about the publication and dissemination plan and how they would recognize the students' contributions.

#### 3.2.2 Component (b): Familiarization with project aims, relevant literature, and dataset

FC gave the students the study protocol to read. Afterward, they attended a meeting with the project leads to discuss the project's aims and research questions. To provide context for code development, each student completed a literature review.

FC was familiar with the entire dataset as she had facilitated all the focus groups and had read through each transcript several times. In planning for the co‐coders' dataset familiarization, FC paired the students, as she felt that direct peer support would be beneficial to students very new to this process. FC then allocated two transcripts to each pair: one transcript from a focus group with supervisors and one from a student focus group. Allocation in this way gave the students broader participant perspectives and aimed to offset some of the time requirements by dividing the dataset. As FC was also going to be co‐coding all the transcripts, she did not feel that each student needed to be familiar with the entire dataset.

Students then individually read through both transcripts allocated to their pair multiple times, noting down thoughts and reflections as they went. After this stage, FC met with all four students as a group to discuss and reflect on the question posed by Vears & Gillam for this stage: “What are these transcripts about?” (p. 117) and additionally “How does this data help to answer our research questions?” Having this early discussion helped guide the students to stay close to the data, within the scope of the research project, and emphasized the importance of having the research questions close at hand.

#### 3.2.3 Component (c): Initial coding

Students then individually completed first‐round coding for both transcripts allocated to their pair. This allowed them to learn and support each other while also having the opportunity to start to develop their own “flavor” of analysis. FC also completed first‐round coding for all four transcripts.

#### 3.2.4 Component (d): Coming to a “shared understanding” of codes

FC met with all four students together over two 1‐h meetings to develop the coding schema. This was achieved by using a shared document for each co‐coder to populate with their codes. Discussions focused on combining similar codes, choosing language that the team felt was most representative of the data, and determining whether there were codes from one analyst that had not been identified by another that needed to be included in the schema. By talking through each of the big‐picture codes, the team achieved a shared understanding of what the codes illustrated in the data.

#### 3.2.5 Component (e): Subsequent coding

The students then proceeded to individually recode one transcript each, using the coding schema developed in Step d. They then individually completed second‐round coding for both transcripts allocated to their pair. FC also completed second‐round coding across all four transcripts. The analysts refined the coding schema across four group meetings led by FC, with students conducting further refined coding between each meeting. At the end of this stage, the group had developed a refined and detailed coding schema.

#### 3.2.6 Component (f): Development of final categories

In this final step of interpretation, FC directed the students to synthesize the content categories and subcategories to answer the research questions as a group. This allowed each student to develop and practice the deep reflective and constructivist skills that this stage requires to tell a relevant and coherent story, while collaborating to incorporate each other's interpretations of the data, and in this way gather the nuance and richness of each analyst's work. The students asked FC to review their first draft, but otherwise completed this step without further supervision. As analysis lead, FC drew on the students' collaborative interpretation when writing up the findings.

### 3.3 Vignette C: Co‐coding with peers

FC collaborated on another project that utilized ICA to analyze data exploring parent experiences of screening high‐risk infants for cerebral palsy. A peer researcher (PR) of FC's invited FC to assist in the analysis as their project conclusion deadline was approaching quickly. The data consisted of 24 semi‐structured interview transcripts, all of which PR had conducted.

#### 3.3.1 Component (a): Planning for collaborative analysis

PR's reasons for using a co‐coding approach to analysis were twofold. Primarily, they wanted to expedite the analysis process without compromising the quality of the research. Secondly, although an experienced qualitative researcher, PR was fairly new to ICA and wanted to co‐code with a peer to ensure their interpretation was not only understandable but also enriched and more nuanced.

PR chose FC because of her experience using ICA, capacity to take on the work within the deadline, and the pair's good working relationship. These choices were conducive to a co‐coding approach. PR also chose only one co‐coder as this struck the right balance for them not only in terms of more than one perspective, but also a limited increase in personnel management.

PR clearly articulated the funded time available to FC, as well as authorship expectations and plans for other knowledge translation strategies. Both analysts were familiar with ICA, so the plan was collaboratively established between FC and PR by looking through the ICA steps and identifying key points of collaboration. In hindsight, further discussion on how the multiple perspectives would be brought together would have been valuable at this stage, as this was not explicit until the end of the process.

#### 3.3.2 Component (b): Familiarization with project aims, relevant literature, and dataset

PR provided FC with a verbal overview of the project and its aims. Although FC was not familiar with the literature related to parent experiences of early screening for cerebral palsy, she was familiar with the literature related to parent experiences of both preterm birth and early intervention for children with cerebral palsy. Again, given the time constraints for this project, PR and FC were comfortable that FC had enough contextual knowledge of the field to proceed with the analysis.

PR was familiar with the entire dataset as they had conducted all the interviews and had read through each transcript several times. PR allocated FC half of the dataset (12 transcripts) and specifically included in this allocation a diversity of parent perspectives. FC then read and familiarized herself with all 12 transcripts.

#### 3.3.3 Component (c): Initial coding

FC and PR each independently completed first‐round coding on three of their 12 allocated transcripts (six transcripts in total).

#### 3.3.4 Component (d): Coming to a “shared understanding” of codes

FC and PR met once to discuss the “big‐picture categories” that had been developed. Although their categories were very similar, they made minor tweaks, with PR making the final decisions as project lead. At the end of this meeting, they had a refined list of codes with a shared understanding of their meanings.

#### 3.3.5 Component (e): Subsequent coding

FC and PR then used this refined list of “big‐picture” codes to independently complete first and second‐round coding on six transcripts each (the three transcripts from Component c) and an additional three.

#### 3.3.6 Component (d): Coming to a “shared understanding” of codes

They met again to discuss the first‐ and second‐round codes and adjust and further refine the coding schema. Again, PR made the final decisions, but FC's perspectives were well represented.

#### 3.3.7 Component (e): Subsequent coding

FC and PR then used this final coding schema to complete first‐ and second‐round coding on the remainder of their allocations of the dataset (a further six transcripts each). They then met three times to collaboratively refine the fine‐grained subcategories.

#### 3.3.8 Component (f): Development of final categories

PR undertook this final step when writing up their publication. FC reviewed their findings and felt that they represented the collaborative interpretation of the dataset.

## 4 RELEVANCE TO GENETIC COUNSELING

Co‐coding in ICA is particularly relevant to the field of genetic counseling for several reasons. First, as we mentioned at the beginning, the practical nature of ICA as an analysis method lends itself well to a large proportion of the research conducted within the field. This is because the goal of a considerable amount of the genetic counseling research undertaken aims to improve practice. Second, there is a large amount of qualitative research in genetic counseling. This not only relates to the nature of the information we are trying to collect to meet the goals of the research but also reflects the value that we, as a profession, place on people's perspectives and experiences. Relatedly, we, as genetic counselors, generally align more with a constructivist view of the world, which means that we also recognize that we each view it through our own lens. This recognition, therefore, suggests that analyzing data through multiple lenses will have value. Lastly, it is a requirement of many (if not most) genetic counseling programs that students undertake a research project. As such, a huge amount of research is being conducted by junior genetic counseling researchers, making mentorship and capacity building through all components of research studies, including data analysis, critical.

## 5 CONCLUSIONS

Due to the constructivist nature of most qualitative research, it is important that researchers are rigorous and transparent about the way(s) in which they have analyzed their data. Having clearly described analytical methods can help researchers both analyze their data methodically and also provide them with a way of representing their processes to others.

This article provides practical guidance for researchers wishing to involve multiple analysts when using inductive content analysis. Involving co‐coders can enhance rigor by supporting more junior researchers (or researchers newer to co‐coding or ICA), bringing diverse perspectives to the interpretation of the data, and dividing the workload. We outline six components of co‐coding that can be tailored depending on the reason for involving co‐coders, who those co‐coders are, and the co‐coders' experiences. We urge consideration of each of the key elements outlined to support best practices in co‐coding in ICA.

## AUTHOR CONTRIBUTIONS

Conceptualization: DFV & FC; Investigation: DFV & FC; Methodology: DFV & FC; Project administration: DFV & FC; Supervision: DFV; Visualization: FC; Writing – original draft: FC & DFV; Writing – review & editing: FL, DFV & FC.

## FUNDING INFORMATION

The authors have no funding to declare.

## CONFLICT OF INTEREST STATEMENT

The authors (Free Coulston, Fiona Lynch, and Danya F. Vears) have no conflicts of interest to declare.

## ETHICS STATEMENT

This manuscript did not involve the collection of participant data and did not require review or approval by an ethical review board.

No human or animal subject were involved and therefore ethics approval is not required.

## Data Availability

Data sharing is not applicable to this article as no new data were created or analyzed in this study.
